# Impacts of a navigation program based on health information technology for patients receiving oral anticancer therapy: the CAPRI randomized controlled trial

**DOI:** 10.1186/s12913-017-2066-x

**Published:** 2017-02-13

**Authors:** Chloé Gervès-Pinquié, Fatima Daumas-Yatim, Benoît Lalloué, Anne Girault, Marie Ferrua, Aude Fourcade, François Lemare, Mario Dipalma, Etienne Minvielle

**Affiliations:** 1 Equipe d’Accueil Management des Organisations de Santé, Ecole des Hautes Etudes en Santé Publique, Avenue du Professeur Léon-Bernard, 35043 Rennes, France; 2 Gustave Roussy, 114 Rue Edouard Vaillant - 1er étage zone B, 94805 Villejuif, France

## Abstract

**Background:**

The emergence of oral delivery in cancer therapeutics is expected to result in an increased need for better coordination between all treatment stakeholders, mainly to ensure adequate treatment delivery to the patient. There is significant interest in the nurse navigation program’s potential to improve transitions of care by improving communication between treatment stakeholders and by providing personalized organizational assistance to patients. The use of health information technology is another strategy aimed at improving cancer care coordination that can be combined with the NN program to improve remote patient follow-up. However, the potential of these two strategies combined to improve oral treatment delivery is limited by a lack of rigorous evidence of actual impact.

**Methods/design:**

We are conducting a large scale randomized controlled trial designed to assess the impact of a navigation program denoted CAPRI that is based on two Nurse Navigators and a web portal ensuring coordination between community and hospital as well as between patients and navigators, versus routine delivery of oral anticancer therapy. The primary research aim is to assess the impact of the program on treatment delivery for patients with metastatic cancer, as measured by Relative Dose Intensity. The trial involves a number of other outcomes, including tumor response, survival, toxic side effects, patient quality of life and patient experience An economic evaluation adopting a societal perspective will be conducted, in order to estimate those health. care resources’ used. A parallel process evaluation will be conducted to describe implementation of the intervention.

**Discussion:**

If the CAPRI program does improve treatment delivery, the evidence on its economic impact will offer important knowledge for health decision-makers, helping develop new follow-up services for patients receiving oral chemotherapy and/or targeted therapy. The process evaluation will determine the best conditions in which such a program might be implemented.

**Trial registration:**

NCT 02828462. Registered 29 June 2016.

## Background

Oral delivery in cancer therapeutics is an emerging issue within the general context of health care cost control and patients’ desire to receive high-quality care at home [1]. This new practice generates needs for better coordination between the treatment stakeholders (patients and care providers) along the care continuum [2–6]. Indeed, in comparison with parenteral chemotherapy (which requires professionals to deliver medication themselves) the use of oral agents allows remote follow-up. Yet, given the toxicity of the medication and its potential side effects, both patients and professionals in the community need to be provided with enhanced information in order to ensure adequate treatment delivery and maximal efficacy. Indeed, high rates of non-adherence to oral anticancer drugs (often associated with a poor Relative Dose Intensity) have been reported in the literature [6, 7], implying adverse impacts on the efficacy and toxicity of the medication - along with an increased use of health care services [8–10].

In line with this need for improved coordination, navigation programs are becoming a major trend in oncology [11, 12]. Their aim is to address the widespread problem of access to (and continuity of) care [13], and these programs often rely on Nurse Navigators who are in charge of improving transitions of care by providing information about cancer (from prevention to treatment), assisting patients with medical paperwork, facilitating patient-provider communication, scheduling appointments, addressing transportation issues and mobilizing patient financial resources [14, 15]. These navigation programs are based on health information technology that has already demonstrated positive impacts on patient self-management with regard to pain and disease symptoms [16–18], as well as patient adherence to oral therapies [19, 20]. However, evidence on the impact of such navigation programs remains scarce. Some programs have proved successful with positive outcomes identified - such as improved patient care, better access to screening tests, and better cancer follow-up rates and timeliness [11, 21–24]. Regarding their economic impact, few economic evaluations of such programs have been conducted for patients having cancer, though not specifically receiving oral therapeutics - with nuanced results on their cost-effectiveness, depending on the perspective adopted for the evaluation [25, 26]. Overall, few studies have provided evidence regarding the impact on quality of care.

Further evaluations relying on randomized controlled trials are therefore needed to provide rigorous evaluation of these programs [27]. The study of the CAPRI program, developed within the Gustave Roussy Comprehensive Cancer Center, aims to address this specific issue. This trial first seeks to measure the impact of a new navigation system combining Health Information Technology (via a web portal) and Nurse Navigators (NN) [28] on treatment delivery for patients with metastatic cancers who are receiving oral chemotherapy and/or targeted therapy. Improved treatment delivery is expected for patients benefiting from the CAPRI program, since this last is supposed to strengthen links between hospital professionals, primary care physicians, pharmacists and nurses in the community.

This paper details the study protocol of a Randomized Controlled Trial (RCT) evaluating the efficacy of a new navigation program named CAPRI. The primary objective is to examine whether it leads to better treatment delivery among metastatic cancer patients treated with oral chemotherapy and/or targeted therapy, in comparison with those receiving only usual care. A secondary objective is to examine how the CAPRI program influences several aspects associated with patient care including tumor response, survival, toxic side effects, quality of life and patient experience. The study also includes both an economic evaluation and a parallel process evaluation.

## Method/design

### Study setting

The proposed Randomized Controlled Trial is a large-scale trial designed to assess the impact of an innovative navigation program denoted CAPRI, that is based on two Nurse Navigators and a web portal and ensures coordination between community and hospital as well as between patients and navigators, within the context of routine delivery of oral anticancer therapies. The broad research aim is to assess (a) the program’s impact on oral therapeutic delivery for patients with metastatic cancer treated with oral chemotherapy and/or targeted therapy, and (b) CAPRI’s effectiveness in terms of the treatment’s toxic side effects, tumor response, Progression Free Survival, Overall Survival, patient quality of life and patient experience, and (c) the economic evaluation of the CAPRI program.

**Fig. 1:**
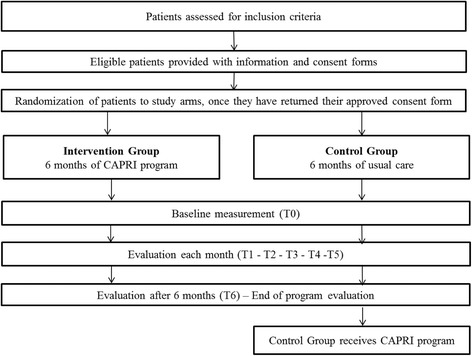
Flow Chart of participant’s progress through the different phases of the study

The study will evaluate the CAPRI’s efficacy in comparison with usual care. The RCT will randomize 1,000 individuals fulfilling inclusion criteria into either the CAPRI group or the control group, for a 6-month period (Fig. 1). Participant outcomes could be assessed at baseline (T0) and each month of intervention, excepted for quality of life (T1 – T6) and patient experience (T6). Control group participants will be offered the intervention when the CAPRI program ends for the intervention group.

### Participants

Recruitment will take place at the Gustave Roussy Comprehensive Cancer Center and will be open to patients with metastatic cancer taking oral chemotherapy and/or targeted therapy. 1,000 patients initiating oral therapeutics will be recruited. Recruitment will be managed by the referring department at Gustave Roussy.

### Eligibility criteria

All participants will be required to speak French and be aged 18 years and older. To be included in the study, they will have to have a performance status, corresponding to the OMS score, of between 0 and 2, and their life expectancy must be of at least 6 months. They must consent to follow-up assessments. Exclusion criteria include being under hormonotherapy only, not having a referring general practitioner (GP), having neither internet nor phone access, and being deprived of liberty.

### Sample size calculation

Given previous research showing Relative Dose Intensity (RDI) varying from 60% to 95% for oral therapeutics in cancer care, considering a Relative Dose Intensity lower than 85% as a signal for reduced survivorship, and considering that a difference of 6% to 20% was found between different treatment options or between different cancer types, we expect a sizeable effect of the CAPRI program on patient RDI (5% difference with the control group) [29–32]. Therefore, 393 individuals per group will be sufficient to detect a difference in RDI between the CAPRI program and usual care, assuming significance is set at 5% (α = 0.05) power at 80% and a standard deviation of 25% for the distribution of mean change. We will include an additional 27% (*N* = 107 per group) to allow for attrition.

### Participant recruitment procedure

Depending on their cancer stage and treatment option, participants will be invited to join the cohort by their referring committee at Gustave Roussy, which will be responsible for their eligibility. In Gustave Roussy, each of these departments is composed of medical doctors, specialized in a specific type of cancer. Those patients considered eligible will be directly offered the CAPRI program by their oncologist, and provided with the participant information pack. Then, the referring oncologist will provide each patient with a consent form, if they are interested. Baseline measurement collection will be conducted only for consenting participants, following randomization. Patients in the intervention group will then meet the Nurse Navigators for an initial appointment, at which they will be given a starter box detailing login details, as well as a guide to using the portal.

### Randomization procedure

In accordance with CONSORT guidelines [33, 34], randomization will occur once participants have completed their consent form. Given the potential difference in adherence and socioeconomic status among the different cancer types [35, 36], stratified randomization might be used in order to minimize these differences between and within groups. This randomization will be done via computer software and completed by an independent researcher (Gustave Roussy randomization department). A randomization number will be allocated to each participant, and results of the randomization will be sent to both the therapist and the nurse navigators. Participants included in the intervention arm will be given immediate access to the CAPRI program, including access to their personalized online portal.

### Intervention: the CAPRI program

Throughout the course of the study, participants from the control group will receive their care as usual, while participants from the intervention group will benefit, for 6 months, from the CAPRI program alongside the usual treatment, comprising a monthly visit to the referring oncologist at Gustave Roussy (mainly for prescription renewal). Both groups will receive oral chemotherapy and/or targeted therapy prescribed at Gustave Roussy, as well as regular check-ups for other treatments.

#### Nurse navigators

Two Nurse Navigators ensure patient follow-up at a distance, via phone interviews and email, from Monday to Friday, during office hours only (9 am to 5 pm). Patients benefit from regular follow-up by phone. Calls will be scheduled as follows: once a week during the first month, once every two weeks from the second to the fourth months and once every three weeks from the fifth month to the end of the study.

Nurse Navigators provide the link between the hospital professionals, patients and primary care professionals (GP, private nurse, pharmacist, etc.) given access to the web portal following patient agreement.

#### Web portal

The web portal provides a unique interface through which healthcare professionals are able to connect with their patients. Each program stakeholder has his/her own login for access to a tailored portal; Table 1 presents its main functionalities.

**Table 1:** Description of the main functionalities of the CAPRI web portal

Functionalities	Description
Messages	Secured message facility for contacting NNs
Follow-up	Tracking of follow-up measures (temperature, weight, pain, appetite) and if necessary, self-reporting of other symptoms
Schedule	Displays and saves appointments on a personal schedule
Address book	Provides access to an address book containing the addresses and phone numbers of professionals enrolled, as well as other useful numbers
Information	Provides access to reference websites providing information about the disease, treatment options and their side effects
Storage	Downloads, saves and files documents relating to patient care (clinical and biological exams, patient records, etc.)
Reminders	Schedules reminders to take medications, arrange appointments, plan exams, and document personal measurements

The web portal provides NNs with a dashboard so that they can monitor the records of all patients enrolled in the program. Following each contact with a patient, NNs can create ‘intervention reports’ detailing what they have done or discussed, and transmit the information to those professionals previously indicated by the patient in question. These professionals can log on the portal to communicate with NNs online and access information regarding the patients they care for (following patient agreement).

The system also generates automatic alerts to patients or NNs. Alerts and patient requests can be generated in various ways:

- automatically, through the web portal, for instance while reporting follow-up measures (if the patient measures are below or above predefined thresholds)
- by the NNs during regular follow-ups
- by a message/call of the patient or the professionals.

The NN evaluates the alert level on the basis of algorithms (NCI-CTCAE classification) and determines the action to implement, in line with navigation algorithms. Depending on level, the NN can give advice, refer the patient to their primary care physician or to a professional at Gustave Roussy, or contact the dedicated services to organize a hospitalization or schedule an appointment for the patient.

### Impact evaluation

#### Primary endpoint

The primary endpoint of the study will be significant change in the delivery of oral treatment, measured by Relative Dose Intensity, which is calculated as the ratio of the dose actually delivered over time to the prescribed dose intensity. Our hypothesis is that, thanks to faster management of treatment side effects, patients in the CAPRI program will demonstrate a significant increase in Relative Dose Intensity, in comparison with those from the control group.

#### Secondary endpoints

Secondary endpoints will be patient adherence to oral anticancer therapy, quality of life (functional scales, symptom scales, global health status and quality-of-life scales), patient experience, tumor response, Progression Free Survival (PFS), Overall Survival (OS) and toxic side effects of the therapy (severity and quantity). We expect that patients in the intervention arm, by comparison with controls, will demonstrate significantly greater improvements in secondary outcomes measures. Demographic, socio-economic and clinical variables will be assessed and considered in order to adjust the study results. These adjustment factors could be used to make stratified group comparisons.

#### Instruments of measurement

Outcomes will be assessed by a Clinical Research Associate (ARC) at baseline, and each month until the end of the program (after 6 months), excepted for patient quality of life (at baseline, after 3 months and at the end of the program) and for patient experience (at the end of the program). Instruments used to collect data from participants, including primary and secondary outcomes, are listed in Table 2.

**Table 2:** Variables and Instruments used for data collection

Measurement tools ARC administered (face to face interview)		Time point			
		At baseline	Each month	After 3 month	After 6 months
Demographics and general medical history	Age, gender, comorbidities	*			
Socioeconomic status	Education, income, employment, family characteristics	*			
Diagnosis	Cancer type, stage	*			
Autonomy	OMS score [55]	*	*	*	*
Treatment Delivery	RDI ratio [30] Morisky questionnaire [56] MEMs (only for targeted therapy) [57]		*	*	
Toxic effects	NCI-CTC-AE (CTCAE v4.0)		*	*	
Overall Survival	OS		*		
Progression Free Survival	PFS		*		
Tumor response	RECIST [58]		*		
Quality of life	EORT QLQ-C30 [59]	*		*	
Patient experience Satisfaction	PACIC [60]				*

### Economic evaluation

The economic evaluation of the CAPRI program will adopt a societal perspective, assessing intervention, medical and non-medical costs. All resources used by patients included in the study will be considered in the frame of a cost-effectiveness study. It is expected that in comparison with usual care, CAPRI intervention will result in cost-effective care with improvement in patients’ adherence to oral anticancer therapy. Cost data will be collected monthly using retrospective self-questionnaires for non-hospital costs. For hospital costs, medico-administrative data will be used. Table 3 presents the resources used to be included in the cost-effectiveness study, and their value units.

**Table 3:** Resources used for the economic evaluation

Resources used		Value units
Medical resources		
Hospital	- Number and length of unplanned hospitalizations (by service department – specify which hospital) - Number and length of planned hospitalizations (by department service – specify which hospital) - Number of unplanned consultations in oncology - Number of planned consultations in oncology -Number of cancelled consultations (by service department – specify which hospital) - Number of consultations in supportive care (by type of supportive care service) -Reduction of supportive care treatment	Stay cost Stay cost Consultation tariff Consultation tariff Consultation tariff Consultation tariff Medication price
Non-hospital	-Number of GP consultations -Number of visits by private nurses -Number of consultations with other paramedical professionals	Consultation tariff Visit tariff Consultation tariff
Non-medical resources		
Transport	- Number of home-to-hospital journeys made (by transport type)	Travel cost
Professional care	-Hours of professional care (by task performed)	Hourly tariff
Informal care	-Hours of informal care	Hourly wage rate
Intervention		
	-Nurse Navigators training -Nurse Navigators wage -Web portal design and implementation -Office equipment (computer, phone, desk)	Hourly wage Hourly wage Web portal invoice + monitoring package Equipment price

### Process evaluation

A parallel evaluation will be conducted to describe both the steps taken to implement the intervention, and the prevailing conditions, which may be important to helping/supporting process implementation. This evaluation will be led as a longitudinal study through quarterly assessments of web portal use by enrolled patients and professionals, alongside evaluations of their satisfaction and needs. Analysis will also include the description of Nurse Navigator activities and specific skills. Data on access to, and use of, the web portal will be extracted from the web portal records. In addition, focus groups with Nurse Navigators will be organized throughout the study. Lastly, semi-structured interviews with patients, relatives and healthcare professionals engaged in the program will be conducted by the research team at the end of the trial. Combining a process evaluation with an outcome evaluation will provide a comprehensive evaluation of the CAPRI program [37]. It will also give important indications as to the sustainability of any improvement in quality achieved [38].

### Data analysis

Appropriate descriptive statistics will allow presentation and comparison of sample characteristics at baseline, and at each evaluation time point. The primary analyses will utilize a between-group design (CAPRI versus control groups) at six time points (each month)). Significant change in treatment delivery will be measured on the RDI ratio, calculated with the Morisky score and/or the Medication Event Monitoring System (MEMs) results. If distribution is normal, a student *t*-test will be used to compare RDI means, as well as secondary outcomes designed as continuous variables. If not, a Mann–Whitney test will be used. Chi2 or Fisher’s test will be used to compare secondary outcomes designed as binary variables. Baseline data will be examined to analyze probability of attrition bias.

### Trial status

The trial began in October 2016 with recruitment to the study. Nurse Navigator training is complete and the web portal’s testing phase is over. It is anticipated that full post-intervention data will be completed by September 2018. Clinical trial number is posted on www.clinicaltrials.gov/.

## Discussion

The proposed study aims to provide a rigorous assessment of the impact of a program - based on Nurse Navigation and Health Information Technology - on oral therapeutic delivery for patients with metastatic cancer.

### Study advantages and disadvantages

Assessing delivery of oral anticancer therapy through measurement of both medication adherence and Relative Dose Intensity needs rigorous methods. Based on the results of previous studies [6], we decided to use the RDI measure using a self-reported questionnaire (Morisky), combined with a MEMs for patients receiving targeted therapy. This two-fold strategy should enable us to avoid the risk of adherence overestimation for psychological reasons (shame, guilt) [4, 39]. It has been presented as the most relevant strategy for measuring medication adherence [40] when the use of objective methods (such as blood sampling) is not possible.

The implementation of a medico-economic evaluation nested within our study is innovative; to our knowledge, evaluations of navigation programs in oncology targeting the treatment phase have been scarce [41]. Economic evaluations are encouraged by experts in this field [42] since they allow us to formulate policy and resource allocation implications, such as the development of financial incentives to improve care coordination. Improving patient adherence to oral anticancer therapy and improving their Relative Dose Intensity via a navigation program combined with Health Information Technology - is challenging for the organization of healthcare. Indeed, improved adherence to oral treatment would probably favor community care for patients with cancer, which would in turn imply reduced unplanned activity for hospitals and increased activity for community cancer care. Changes in patient follow-up will generate a need to better inform (train) community care professionals (which could be supported by a navigation program offering a web portal for professionals, as CAPRI does) [43] and adaptation of the payment system to this new care organization, which requires increased coordination between primary and secondary care [44].

The fact that it is designed as a randomized controlled trial is another of our study’s assets. Few high-quality studies provide an evaluation of coordination programs in cancer care, especially combining both Nurse Navigation and Health Information Technology [43–47]. RCT has been considered the gold standard for evaluating organizational interventions [48]. Portela showed that RCTs are relevant for interventions that are expected to be widespread, because of their validity and their evidence-based results [45]. However, this type of trial is based on the biomedical model that assumes a linear causation, which may be irrelevant when considering organizational interventions [45, 48]. Indeed, the effects of such interventions could be attributed to the influence of psychological or social confounders, especially in barely-controlled environments. Nielsen suggested that RCT for organizational interventions should be complemented by another type of evaluation, capable of detailing the implementation processes of an intervention in addition to its ‘direct’ effects [37, 49].

Combining RCT with process evaluation, as we will do in our study, may lead to improved understanding of the impact of such interventions, since it may bring knowledge of the enabling and impeding factors influencing their implementation. [44, 49–51]. It could explain how, why and when organizational interventions generate improvement, taking implementation duration into account [52–54] more effectively than RCTs.

## Abbreviations

ARC: Clinical Research Associate
CPP: Comité de Protection des Personnes
GP: General Practitioner
MEMs: Medication Event Monitoring System
NN: Nurse Navigators
OS: Overall Survival
PFS: Progression Free Survival
RCT: Randomized Controlled Trial
RDI: Relative Dose Intensity
